# A Curious Effect of Acid Irritation of the Kidneys

**Published:** 1846-06

**Authors:** Robert Dick


*A curious effect of Acid Irritation of the Kidneys.* By Robert Dick, M. D.—A gentleman, about eight and thirty, has more than once complained to me of symptoms caused by the use of acerb articles of food. Within from four to eight hours after the unguarded employment of such food, he had already informed me that pains or twinges of a peculiar kind affected his thighs, sometimes extending to his calves. These pains resemble now sharp pricking by a pin; now something akin to, but not *quite* resembling, a very severe *pinch* by the finger and thumb of another person. Meantime the kidney itself was not the seat of any pain, though, from preternatural acidity of the urine, there was ardor urinæ. On the following day, however, there was usually pain, on either side of the lumbar spine, indicating renal irritation. The treatment which I suggested, and which was always effectual on such occasions, was half a dram of carbonate of potass, largely diluted, to which were added from two to five grains of nitrate of potass, to direct the fluid to the kidneys, and carry out the acid secretions going on or completed there, and the presence of which were the cause of the suffering of these organs.

The last attack is of so curious a sort, that I am desirous of recording it. The patient, whether from some structural change in the heart or liver (for he has suspicious symptoms in both organs,) or from a less formidable cause, is a good deal annoyed with a loaded state of the intestinal mucous membrane, leading to a tumid state of the rectum, occasional prolapsus and sanguineous exhalation from the everted surface. By my suggestion he has on these occasions used cautiously a cooling sub-acid diet, such as grapes, stewed apples, stewed pears, stewed rhubarb, cider, claret. These, along with small doses of the milk of sulphur, and the bitartrate of potass, have relieved the protrusion of the bowel, without inducing derangement of the kidney. But last week, this gentleman, suffering more than usual from the tumid state of the rectum, took, on each of two succeeding days, a dozen of the rather sour apples used for stewing, and though these were cooked and sweatened, he experienced, on the nights of the second and third day, the following singular affection.

On the afternoon of that day, he noticed that the quantity of urine was diminished, and that there was a soreness and fulness on either side of the lumbar spine; but from neither of these causes did he suffer any direct pain or inconvenience of the slightest consequence. But about eight o'clock at night, he began to feel at the inner and lower extremity of the right femur, and just between that and the patella, a pain, which, resembling at first the usual one, like the prick of a pin, gradually increased in vehemence, till it became all but intolerable. He could compare it, he said, to nothing but flashes of intense pain, and he tells me he could give me a diagram of this pain. He might compare it, he said, to a star and radii of about an inch and a half square; the pains "corruscating," so to speak, from a central point, to the extent above named. Sometimes the sensation was somewhat of a tearing kind, and went deeper, resembling somewhat, so far as I could comprehend, the lacerating pain experienced in gout. It was such as totally to prevent sleep for many hours, but it did not cause any febrile excitement, nor did it terminate in perspiration either of the part, or general. It left him towards morning. He was entirely free from pain everywhere else, with the exception of the slight uneasiness in the renal regions already indicated. His urine the next morning, and during the next day was strongly acid.

I attributed the affection to irritation of the kidney, and of course regarded the pain at the knee, as analogous to those pains, seemingly situated in the fingers and toes of persons who have lost limbs by amputation; the pains being really seated, if we may use the word, at the origin of the nerves, going to the lost parts. The irritation of the kidneys had, doubtless, in some way, extended to the spinal cord, or to the roots of the lumbar nerves.

I ordered diluents and a general laxative, and having noticed in some similar affections a tendency to periodicity, I suggested an opiate at bed time. The propriety of this was shewn by the recurrence of pain that night, with such considerable severity, that my patient assured me, that but for the opiate he would have suffered no less than on the preceding night. As it was, he suffered less; still he suffered not a little, and though he dozed he did not enjoy sound sleep. A repetition of the opiate, however, secured this on the next night, and removed entirely the attack.—*Lond. Med. Gaz.*

